# Caspases and p38 MAPK Regulate Endothelial Cell Adhesiveness for Mesenchymal Stem Cells

**DOI:** 10.1371/journal.pone.0073929

**Published:** 2013-09-12

**Authors:** Irina A. Potapova, Ira S. Cohen, Sergey V. Doronin

**Affiliations:** Department of Physiology and Biophysics, Institute of Molecular Cardiology, Stony Brook University, Stony Brook, New York, United States of America; IISER-TVM, India

## Abstract

Mesenchymal stem cells natively circulating or delivered into the blood stream home to sites of injury. The mechanism of mesenchymal stem cell homing to sites of injury is poorly understood. We have shown that the development of apoptosis in endothelial cells stimulates endothelial cell adhesiveness for mesenchymal stem cells. Adhesion of mesenchymal stem cells to apoptotic endothelial cells depends on the activation of endothelial caspases and p38 MAPK. Activation of p38 MAPK in endothelial cells has a primary effect while the activation of caspases potentiates the mesenchymal stem cell adhesion. Overall, our study of the mesenchymal stem cell interaction with endothelial cells indicates that mesenchymal stem cells recognize and specifically adhere to distressed/apoptotic endothelial cells.

## Introduction

Natively circulating or systemically delivered mesenchymal stem cells (MSCs) home to sites of injury and facilitate tissue repair. Tissue repair is initiated by inflammation that develops within a few hours after an injury. During this time, neutrophils home to the site of injury causing attraction of monocytes and a massive release of inflammatory factors and free radicals. Cell death and concomitant accumulation of macrophages lead to the resolution of inflammation followed by fibroplasia and tissue remodeling.

Some data suggest that the most effective time for MSC homing is 4–10 days after an injury [1], [2]. This time period coincides with the accumulation of macrophages and the resolution of inflammation. MSC homing during the development of inflammation is relatively inefficient [2], [3]. Similar to leukocytes, MSCs display coordinated rolling behavior on endothelial cells (ECs) activated by inflammatory factors [4], however, they poorly adhere to immobilized endothelial adhesion molecules [5] and ECs activated by inflammatory factors [6], [7]. This deficiency in adhesion to activated ECs can be traced to progressive loss of CXCR4 and other chemokine receptors by MSCs after removal from the bone marrow [7]–[9]. Transfection with lentiviruses harboring the CXCR4-gene [3] or the upregulation of CXCR4-expression by culturing in a 3D-microenvironment [7] facilitates earlier homing of MSCs suggesting that CXCR4-mediated activation of integrins and cell motility might play a role in the regulation of MSC homing to the inflamed tissue [3], [10].

Despite the loss of affinity to ECs activated by inflammatory factors, MSCs are not completely homing impaired. Clinical studies [1], animal models [2], [3] and *in vitro* assays [6], [7] argue that in addition to CXCR4-dependent adhesion MSCs might have alternative mechanisms of adhesion to ECs. For instance, MSCs might recognize and adhere to distressed/apoptotic ECs [6]. MSC adhesion to ECs correlates with the inhibition of endothelial mitochondrial transmembrane potential suggesting that the intrinsic apoptotic pathways of ECs might play a role in the regulation of MSC adhesion [6]. In this article we discuss the role of stress-activated and apoptotic pathways of ECs in the regulation of EC adhesiveness for MSCs.

## Materials and Methods

### Reagents

Recombinant human TNF-α, recombinant human IL-1ß, actinomycin D and cycloheximide were purchased from Sigma-Aldrich (St. Louis, MO). P38 protein kinase inhibitor trans*-*4-[4-(4-Fluorophenyl)-5-(2-methoxy-4-pyrimidinyl)-1H-imidazol-1-yl]-cyclohexanol (SB239063), lipoxygenase inhibitor baicalein and phospholipase inhibitor PACOCF3 were from Tocris Biosciencies. Caspase inhibitor Z-VAD-FMK was purchased from R&D Systems. Phospho-p38 MAPK (Thr180/Tyr182) antibody and p38 MAP kinase antibody were from Cell Signaling Technology.

### Cell Culture

Human MSCs and human umbilical vein endothelial cells (HUVECs) were purchased from Lonza. MSCs were cultured in chemically defined mesenchymal stem cell medium (MSCGM-CD™ BulletKit, Lonza). HUVECs were cultured in supplemented EBM-2 basal medium (EGM-2 Bullet Kit, Lonza). Cells were maintained at 37°C in a humidified atmosphere of 5% CO_2_.

### Immunofluorescent Staining and Flow Cytometric Analysis of Surface Antigen Expression in HUVECs

HUVECs were dissociated from a monolayer with Enzyme Free Cell Dissociation Solution (Millipore), resuspended in the flow cytometry buffer consisting of 2% bovine serum albumin (Sigma) and 0.1% sodium azide (Sigma-Aldrich) in Dulbecco’s phosphate buffered saline (PBS; Sigma). Cells (2×10^5^) were stained using the manufacturer’s suggested concentrations of fluorochrome-conjugated monoclonal antibodies for 30 min at room temperature in the dark. Antibodies to human CD31 (PECAM-1), CD54 (ICAM-1) and CD106 (VCAM-1) were from BD Biosciences. After the staining, cells were washed with 5 ml of the flow cytometry buffer and resuspended in the flow cytometry buffer containing 1% paraformaldehyde (Electron Microscopy Sciences). Background staining was assessed by incubation of cells with mouse fluorochrome- and isotype-matched immunoglobulins (isotype controls). Flow cytometric analysis of HUVECs was performed by analyzing 5,000 events on a FACScan flow cytometer (Becton–Dickinson). A CellQuest™ software package was used to process flow cytometry data. The cellular debris was assessed on the basis of forward and right angle scattering analysis and excluded from future analysis by the CellQuest™ software package.

### Cell Adhesion Assay

MSCs were labeled with 1,1′-dioctadecyl-3,3,3′,3′-tetramethylindocarbocyanine perchlorate (Invirtogen) according to the manufacturer’s recommendations, dissociated with trypsin-EDTA solution (Lonza) and washed with Hank’s balanced salt solution (HBSS, Sigma) containing 0.1% bovine serum albumin (BSA). Confluent HUVECs were grown in supplemented EBM-2 medium on a 96-well cell culture clear-bottom black plate (Corning Incorporated Life Sciences). Before the adhesion assay HUVECs were washed with 100 µl HBSS supplemented with 0.1% BSA. Adhesion assay was initiated by the addition of 10,000 MSCs suspended in 100 µl HBSS supplemented with 0.1% BSA to HUVECs in 100 µl HBSS supplemented with 0.1% BSA. Cells were incubated for 10 minutes at 37°C in a humidified atmosphere of 5% CO_2_ and wells were washed 3 times with 200 µl HBSS supplemented with 0.1% BSA. Cell fluorescence was recorded on a POLARstar OPTIMA microplate reader (BMG Labtech) before and after the wash. Wells without the addition of MSCs were used to measure background fluorescence. Percentage of adherent MSCs was calculated as a ratio of fluorescence signals before and after removal of unbound MSCs after subtraction of the background fluorescence from both values.

### Mitochondrial Transmembrane Potential Assay

Confluent HUVECs were grown on a 96-well cell culture clear-bottom black plates in supplemented EGM-2 medium. Before the assay, cells were washed with PBS and loaded with JC-1 dye for 30 min at 37°C and 5% CO_2_ as recommended by the manufacturer (MitoCapture Apoptosis Detection Kit, BioVision). JC-1 dye accumulation in mitochondria was recorded on a POLARstar OPTIMA microplate reader using red fluorescence with excitation/emission wavelengths at 544/615 nm. JC-1 release to the cytosol was measured in green fluorescence spectrum with excitation/emission wavelengths at 488/590 nm. Mitochondrial membrane permeability was calculated as the ratio of red to green fluorescence.

### Caspase Activity Assay

Caspase-3/7 activation was measured using the SensoLyte AFC caspase profiling kit (AnaSpec). HUVECs were lysed according to the manufacturer’s recommendations, and hydrolysis of fluorogenic caspase substrates was recorded on a POLARstar OPTIMA microplate reader at 37°C for 0–60 minutes with excitation/emission wavelengths of 488/590 nm. Caspase activity was calculated as the rate of substrate hydrolysis.

### P38 MAPK Activation Assay

HUVECs were lysed in 25 mM Tris-HCL, pH 7.5, 1% Triton, 0.5% NP-40, 1 mM EDTA buffer at room temperature for 5 minutes. Protein concentration was determined by Bradford assay (Bio-Rad) and 5 µg of total protein were resolved on SDS-page 10% precast Criterion gel (Bio-Rad). After protein transfer to nitrocellulose membrane (Bio-Rad) p38 MAPK was stained with phospho-p38 MAPK (Thr180/Tyr182) antibody and total p38 MAP kinase antibody. Stained proteins were visualized using a secondary goat anti-rabbit peroxidase conjugated antibody.

## Results

### MSC Adhesion to HUVECs

**Figure 1:**
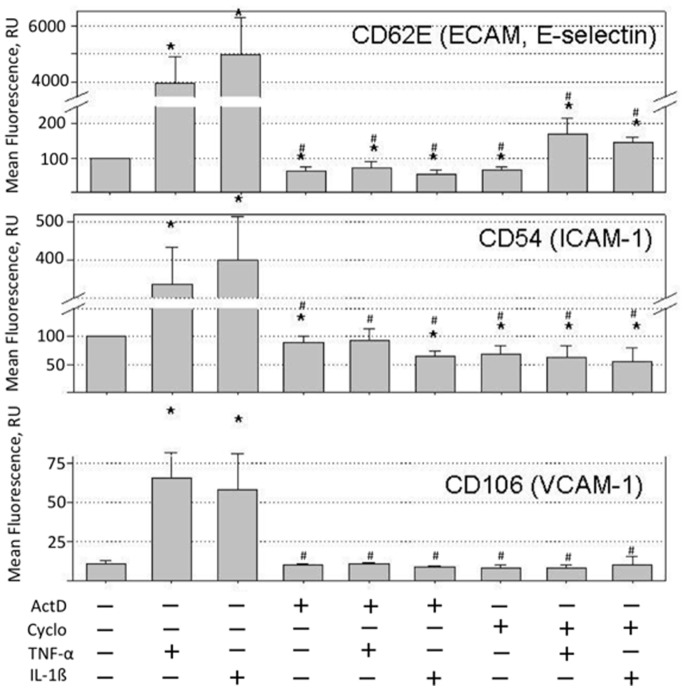
Expression of E-selectin, ICAM-1 and VCAM-1 on the surface of HUVECs treated with TNF-α or IL-1β. HUVECs were treated for 4 hours at 37°C and 5% CO_2_ with 10 ng/ml TNF-α or 10 ng/ml IL-1β in supplemented EGM-2 medium in the absence and presence of 20 µg/ml cycloheximide (Cyclo) or 10 µg/ml actinomycin D (ActD). After the treatment, cells were stained with fluorescein conjugated E-selectin, ICAM-1 or VCAM-1 antibody and analyzed in a flow cytometer. TNF-α upregulated the cell surface expression of E-selectin, ICAM-1 and VCAM-1 40-, 3- and 6-fold, respectively. IL-1ß upregulated the cell surface expression of E-selectin 50-fold, and the cell surface expression of ICAM-1 and VCAM-1 was stimulated 4- and 5.3-fold, respectively. Cycloheximide and actinomycin D inhibited the stimulation of the cell surface expression of E-selectin, VCAM-1 and ICAM-1 by TNF-α or IL-1ß. Data shown are the averages and standard deviations of four independent experiments. Asterisks mark statistically significant changes (p-value <0.05) in comparison with untreated HUVECs. Pound keys mark statistically significant downregulations (p-value <0.05) of the cell surface expression of E-selectin, ICAM-1 and VCAM-1 in comparison with HUVECs treated with TNF-α or IL-1β.

ECs are not pro-adhesive in the basal state and get activated after exposure to inflammatory factors. Activation of ECs by inflammatory factors involves *de novo* synthesis of endothelial adhesion molecules and is blocked in the presence of inhibitors of RNA or protein synthesis. Data in Figure 1 show the expression of E-selectin, ICAM-1 and VCAM-1 on the cell surface of HUVECs treated with 10 ng/ml IL-1ß or 10 ng/ml TNF-α for 4 hours. IL-1ß and TNF-α induced robust expression of E-selectin, ICAM-1 and VCAM-1 on the surface of ECs that was inhibited in the presence of 10 µg/ml actinomycin D, an inhibitor of RNA synthesis, or 20 µg/ml cycloheximide, an inhibitor of protein synthesis (Fig. 1).

**Figure 2:**
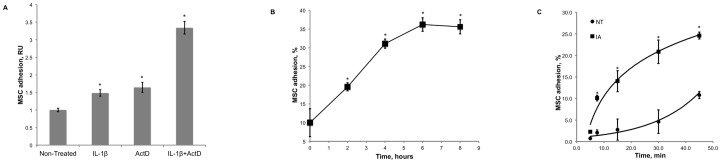
MSC adhesion to HUVECs. HUVECs were treated at 37°C and 5% CO_2_ with 10 ng/ml IL-1ß, 10 µg/ml actinomycin D (ActD) or 10 ng/ml IL-1ß in the presence of 10 µg/ml actinomycin D for 0–8 hours in supplemented EGM-2 medium. ***Panel A*** shows MSC adhesion to HUVECs treated with IL-1ß, actinomycin D or IL-1ß in the presence of actinomycin D for 4 hours. IL-1ß stimulated the MSC adhesion 1.5-fold. Actinomycin D stimulated the MSC adhesion 1.6-fold. A mixture of IL-1ß and actinomycin D stimulated the MSC adhesion 3.5-fold. Data shown are the averages and standard deviations of five adhesion assays. Asterisks mark statistically significant changes (p-value <0.05) in the MSC adhesion in comparison with the adhesion to untreated HUVECs. ***Panel B*** shows the changes in HUVEC adhesiveness for MSCs during the treatment with IL-1ß in the presence of actinomycin D for 0, 2, 4, 6 and 8 hours. Data shown are the average percentages of adherent MSCs (n = 4) after 10 minutes of incubation with HUVECs. Error bars represent standard deviations. Asterisks mark statistically significant changes (p-value <0.05) in the MSC adhesion in comparison with the adhesion to untreated HUVECs. ***Panel C*** shows the time courses of MSC adhesion to untreated HUVECs (NT) and to HUVECs treated with IL-1ß in the presence of actinomycin D (IA) for 4 hours. Data shown are the averages and standard deviations of four adhesion assays. Asterisks mark the time points when the MSC adhesion was statistically different (p-value <0.05) from MSC adhesion to untreated HUVECs.

At given experimental conditions IL-1ß and actinomycin D stimulated MSC adhesion to HUVECs 1.5-fold and 1.6-fold, accordingly (Fig. 2A). A mixture of IL-1ß with actinomycin D stimulated MSC adhesion 3.5-fold. Adhesion of MSCs to HUVECs activated with IL-1ß in the presence of actinomycin D was time dependent and required 6 hours to fully develop (Fig. 2B). The time course of MSC adhesion to HUVECs in the basal state and to HUVECs treated with a mixture of IL-1ß and actinomycin D is shown on Figure 2C. Treatment of HUVECs with IL-1ß in the presence of actinomycin D accelerated the MSC adhesion.

The pattern of MSC adhesion to HUVECs activated with IL-1ß was different from that reported for the adhesion of neutrophils or monocytes to HUVECs activated with IL-1ß or TNF-α [11], [12]. Although MSC adhesion to HUVECs was stimulated by IL-1ß, the inhibition of the *de novo* synthesis of endothelial adhesion molecules by actinomycin D did not inhibit but rather potentiated the adhesion of MSCs.

### MSC Adhesion to HUVECs Correlates with Mitochondrial Failure in Endothelial Cells

**Figure 3:**
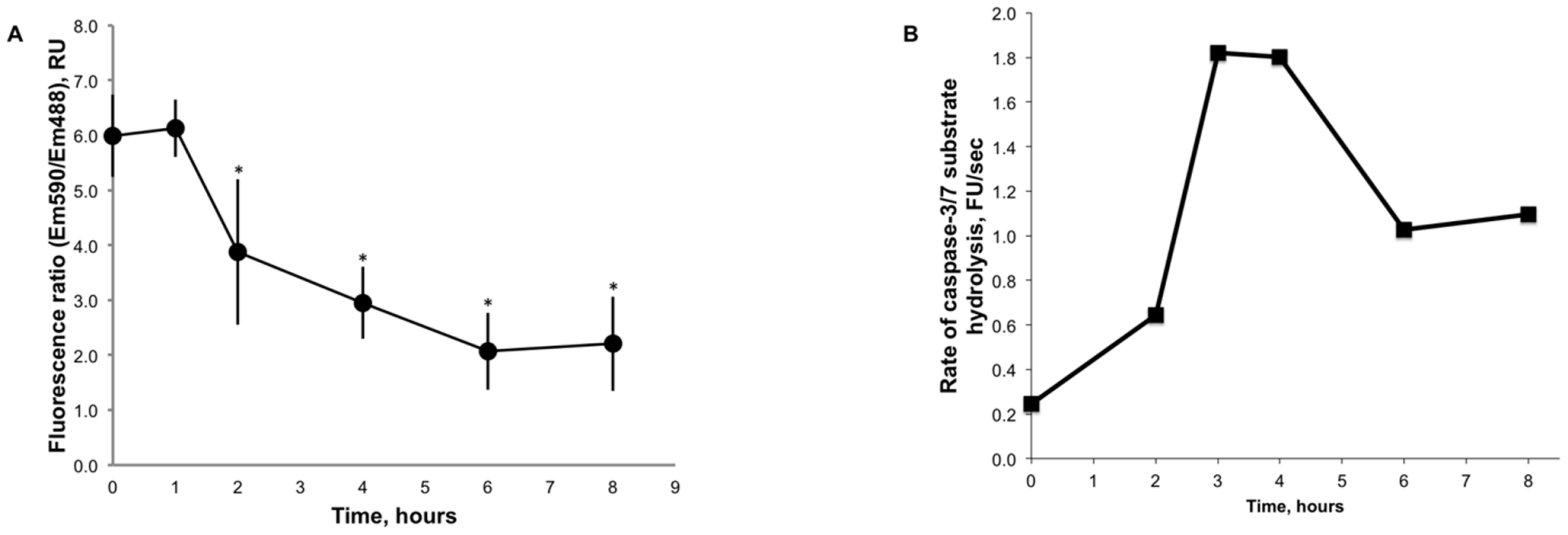
Endothelial apoptosis. HUVECs were treated at 37°C and 5% CO_2_ with 10 ng/ml IL-1ß in the presence of 10 µg/ml actinomycin D for 0–8 hours in supplemented EGM-2 medium. ***Panel A*** shows the inhibition of mitochondrial transmembrane potential by a mixture of IL-1ß with actinomycin D. Mitochondrial transmembrane potential was assayed as the ratio of JC-1 dye located in mitochondria (emission wavelength 590 nM) to cytosol bound JC-1 (emission wavelength 488 nM). Data shown are the averages and standard deviations of four assays. Asterisks mark statistically significant changes (p-value <0.05) in JC-1 dye distribution. ***Panel B*** shows caspase-3/7 activation in HUVECs treated with IL-1ß in the presence of actinomycin D for 0, 2, 4, 6 and 8 hours. Caspase activity was determined as the rate of hydrolysis of caspase specific fluorogenic substrates. Data shown are the averages of two independent assays.

Data in Figure 3A show the distribution of JC-1 dye between the cytosol and mitochondria of HUVECs treated with IL-1ß in the presence of actinomycin D. Within 8 hours of incubation mitochondria of HUVECs was increasingly permeabilized resulting in the depolarization of mitochondrial membrane and the release of JC-1 into the cytosol. Development of apoptosis in HUVECs treated with IL-1ß in the presence of actinomycin D was confirmed by the analysis of caspase activity in cellular lysates. Caspase-3/7 was activated in HUVECs treated with IL-1ß in the presence of actinomycin D within 0–8 hours (Fig. 3B).

**Figure 4:**
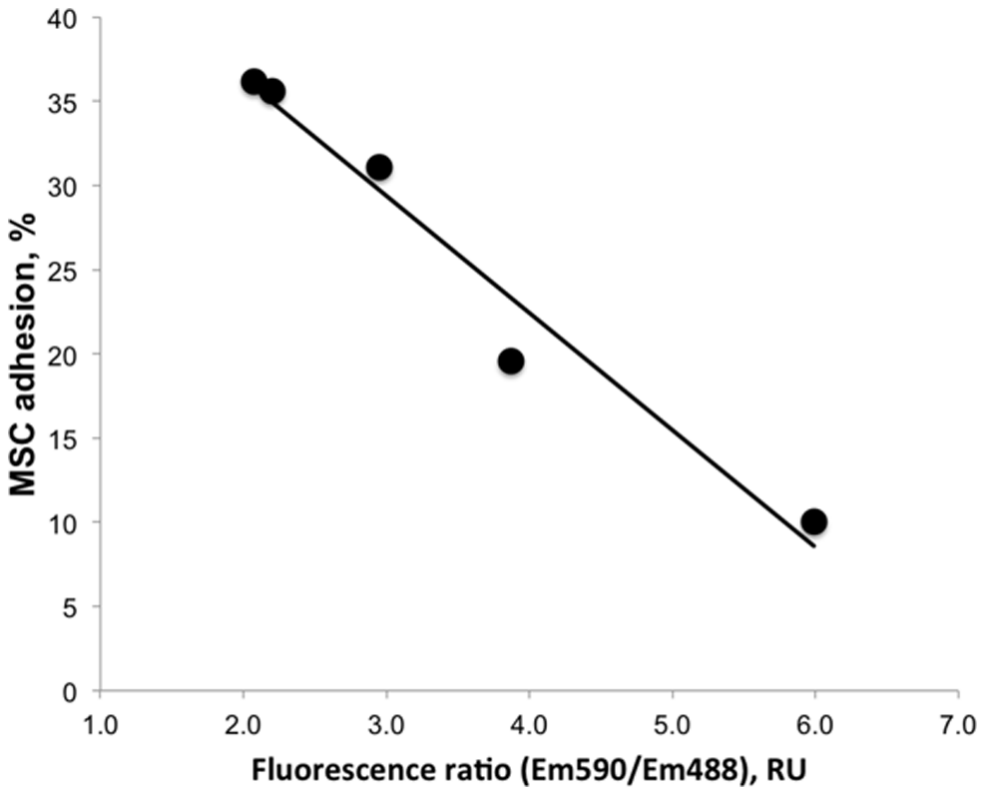
Correlation of MSC adhesion to HUVECs with the inhibition of mitochondrial transmembrane potential of ECs. Data from Figures 2B and 3A were used to estimate Pearson’s correlation between the MSC adhesion and the inhibition of mitochondrial transmembrane potential of HUVECs. MSC adhesion showed a strong correlation (R = 0.98, P-value = 0.003) with the inhibition of mitochondrial transmembrane potential of ECs.

We used the data of the time course of HUVEC activation by a mixture of IL-1ß and actinomycin D (Fig. 2B) and the data of the inhibition of mitochondrial transmembrane potential (Fig. 3A) to correlate the MSC adhesion with the inhibition of mitochondrial function in HUVECs. The results of the analysis are shown on Figure 4. The MSC adhesion strongly correlated with the inhibition of mitochondrial transmembrane potential in HUVECs (R = 0.98, P-value = 0.003).

### Caspase Activation in Endothelial Cells Regulates the MSC Adhesion

**Figure 5:**
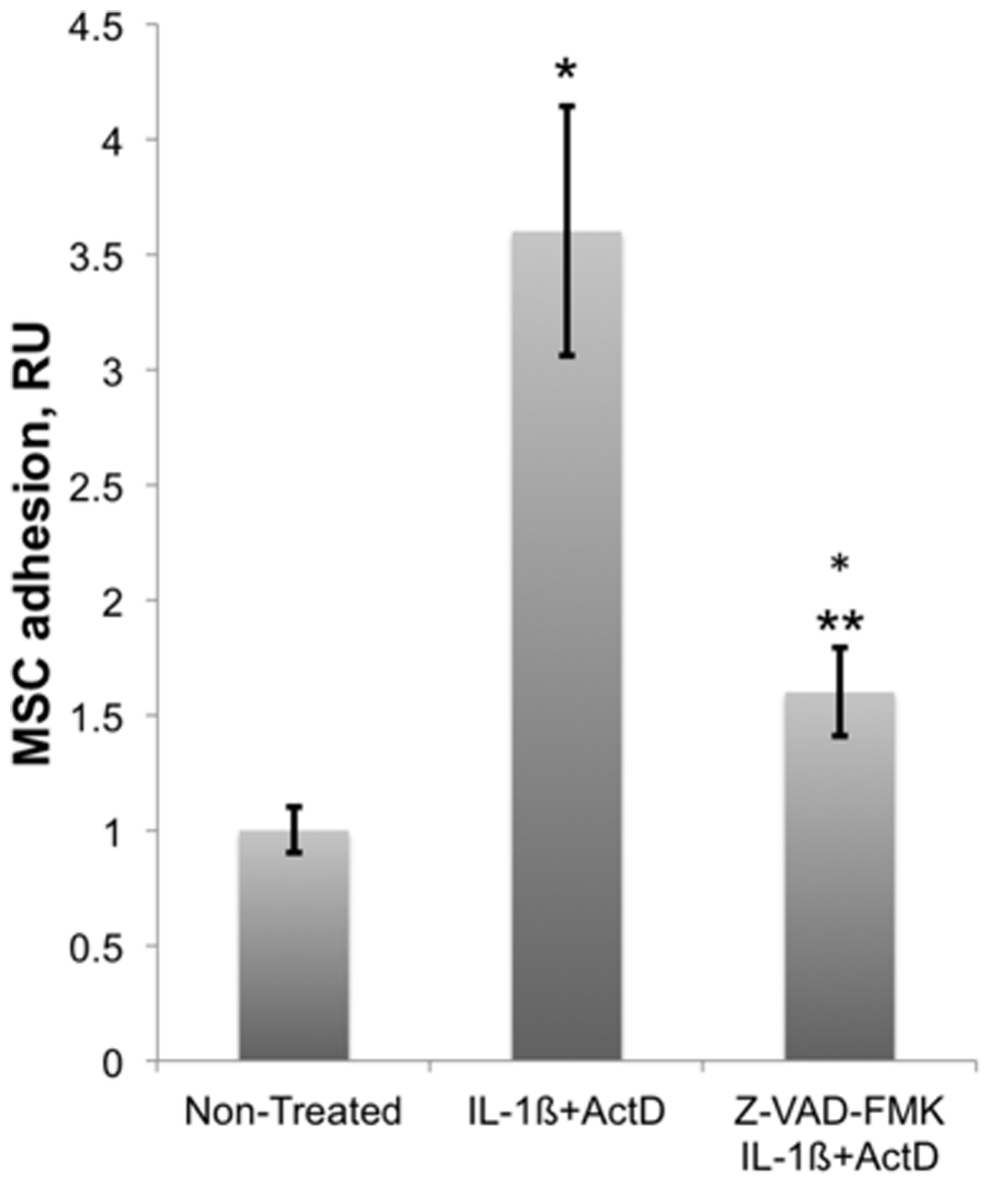
Effect of a caspase inhibitor, Z-VAD-FMK, on MSC adhesion to HUVECs. HUVECs were treated at 37°C and 5% CO_2_ with a mixture of 10 ng/ml IL-1ß and 10 µg/ml actinomycin D (ActD) in the presence and absence of 50 µM Z-VAD-FMK for 4 hours in supplemented EGM-2 medium. Data shown are the averages and standard deviations of five adhesion assays. Stimulation of the MSC adhesion with a mixture of IL-1ß and actinomycin D was reduced from 3.6- to 1.6-fold in the presence of Z-VAD-FMK. Asterisks mark statistically significant changes (p-value <0.05) in the MSC adhesion in comparison with the adhesion to untreated HUVECs. Double asterisk marks statistically significant inhibition (p-value <0.05) of the MSC adhesion by Z-VAD-FMK in comparison with MSC adhesion to HUVECs treated with IL-1ß in the presence of actinomycin D.

**Figure 8:**
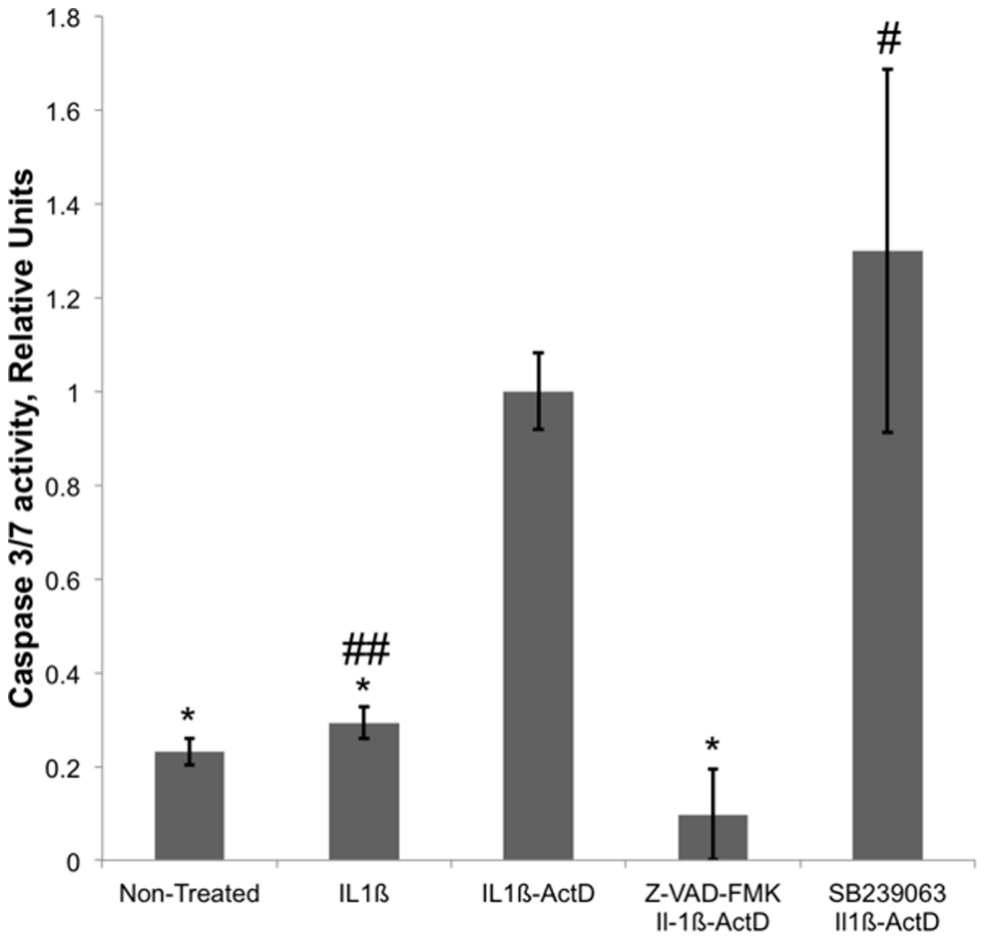
Effect of SB239063 and Z-VAD-FMK on caspase-3/7 activity in HUVECs. Cells were treated for 4 hours at 37°C and 5% CO_2_ with 10 ng/ml IL-1ß or a mixture of 10 ng/ml IL-1ß and 10 µg/ml actinomycin D (ActD) in the presence and absence of 50 µM SB239063 or 50 µM Z-VAD-FMK. Caspase activity was determined as the rate of hydrolysis of caspase-3/7 specific fluorogenic substrate by HUVEC lysates. Data shown are the averages and standard deviations of four independent assays after normalization on caspase-3/7 activity in the cells treated with a mixture of IL-1ß and actinomycin D. IL-1ß had no statistically significant effect on caspase-3/7 activity in HUVECs (p-value >0.05, marked with a double pound key). Caspase-3/7 activity in the non-treated cells or the cells treated with IL-1ß was five-fold lower than the activity in the cells treated with a mixture of IL-1ß and actinomycin D (p-value <0.05, marked with asterisks). Z-VAD-FMK inhibited caspase-3/7 activity in HUVECs treated with IL-1ß and actinomycin D ten-fold (p-value <0.05, marked with an asterisk). SB239063 had not statistically significant effect on caspase-3/7 activity in HUVECs treated with IL-1ß and actinomycin D (p-value >0.05, marked with a pound key).

In order to further investigate the correlation between the MSC adhesion and the mitochondrial failure in HUVECs we studied the effects of a caspase inhibitor Z-VAD-FMK on the MSC adhesion. Data in Figure 5 show that the presence of pan-caspase inhibitor Z-VAD-FMK (50 µM) during the treatment of HUVECs with IL-1ß and actinomycin D inhibited the stimulation of the MSC adhesion from 3.6-fold to 1.6-fold. At this concentration of Z-VAD-FMK the activity of caspase-3/7 in HUVECs was inhibited tenfold (Fig. 8).

### Inhibition of Lipoxygenase and Phospholipase A2 did not Affect MSC Adhesion to ECs

**Figure 6:**
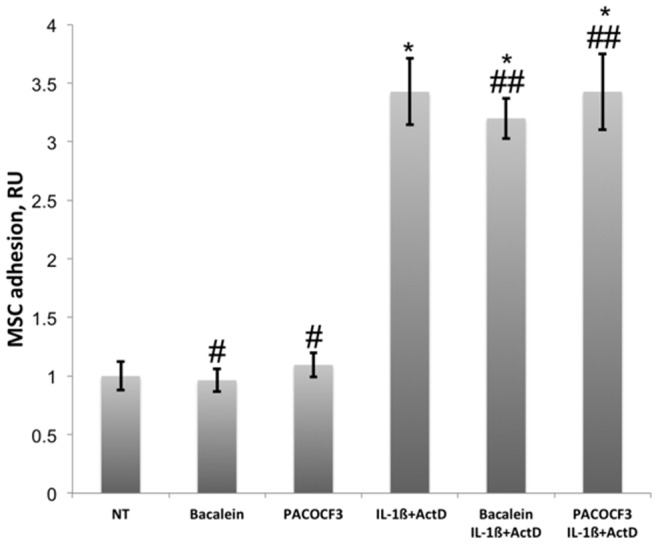
Effects of Baicalein and PACOCF3 on MSC adhesion to HUVECs. HUVECs were treated at 37°C and 5% CO_2_ with a mixture of 10 ng/ml IL-1ß and 10 µg/ml actinomycin D (ActD) in the absence and presence of 50 µM baicalein or 10 µM PACOCF3. Baicalien and PACOCF3 had no statistically significant effects on MSC adhesion to HUVECs in the basal state or to HUVECs treated with IL-1ß in the presence of actinomycin D. Pound keys mark statistically insignificant effect of baicalien or PACOCF3 on MSC adhesion to HUVECs in basal state (n = 5, p-value >0.05). Double pound keys mark statistically insignificant effect of biacalein or PACOCF3 on MSC adhesion to HUVECs treated with a mixture of IL-1ß and actinomycin D (IL-1ß+ActD, n = 5, p-value >0.05) in comparison with MSC adhesion to HUVECs treated with IL-1ß in the presence of actinomycin D. Single asterisks mark statistically significant difference in MSC adhesion in comparison with the adhesion to HUVECs in basal state (n = 5, p-value <0.05).

Depolarization of mitochondrial membrane disrupts the electron transport chain of mitochondria leading to accumulation of peroxides that oxidize non-saturated fatty acids [13]. Oxidized derivatives of arachidonic acid participate in the regulation of EC adhesiveness for monocytes [14], [15]. Release of these mediators of monocyte adhesion depends on the activity of phospholipase A2 and lipoxygenase. We tested the effects of baicalein, an inhibitor of lipoxygenase, and PACOCF3, an inhibitor of phospholipase A2, on MSC adhesion to HUVECs treated with IL-1ß in the presence of actinomycin D. Baicalein at concentrations of 50 µM and 10 µM PACOCF3, respectively, did not inhibit the activation of HUVECs by a mixture of IL-1ß and actinomycin D and did not inhibit MSC adhesion to ECs (Fig. 6).

### p38 MAPK of Endothelial Cells Regulates the MSC Adhesion

**Figure 7:**
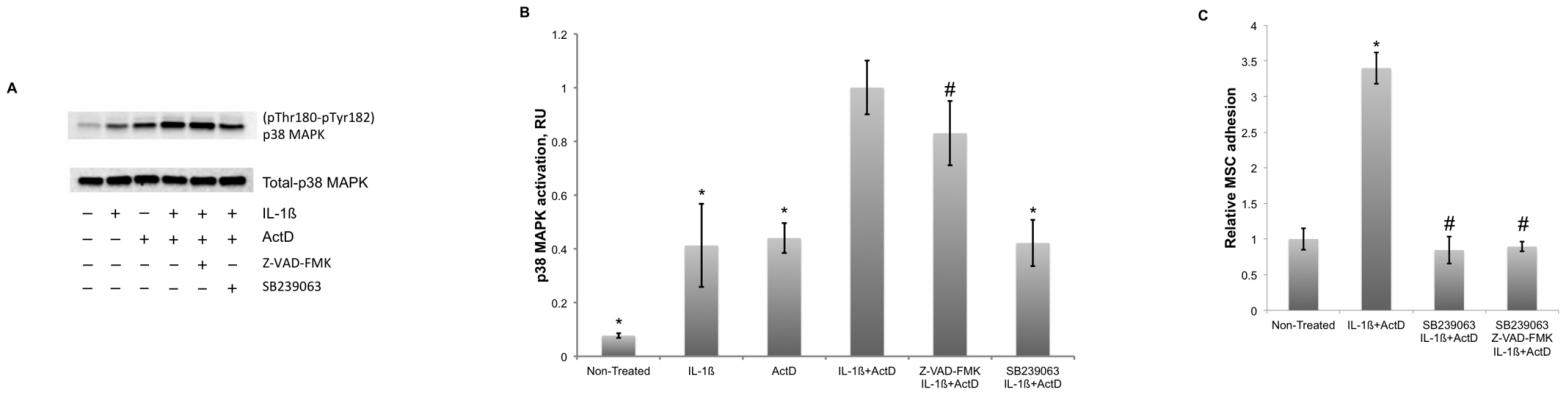
P38 MAPK phosphorylation in HUVECs and its role in the regulation of the MSC adhesion. P38 MAPK kinase activation was studied in HUVECs treated for 4 hours at 37°C and 5% CO_2_ in supplemented EGM-2 medium with 10 ng/ml IL-1ß, 10 µg/ml actinomycin D (ActD) or a mixture of 10 ng/ml IL-1ß and 10 µg/ml actinomycin D (ActD) in the absence and presence of 50 µM Z-VAD-FMK or 50 µM SB239063. ***Panel A*** shows the Western Blot analysis of p38 MAPK phosphorylation at Thr-180 and Tyr-182 in untreated HUVECs and HUVECs treated with IL-1ß, actinomycin D or a mixture of IL-1ß and actinomycin D in the presence and absence of Z-VAD-FMK or SB239063. Staining for total p38 MAPK was performed to normalize the signals from phosphorylated p38 MAPK. ***Panel B*** shows quantitative analysis of p38 MAPK activation. Western blot images were acquired on a Kodak Image Station 440 and processed with supplemented software. P38 MAPK phosphorylation in untreated HUVECs and HUVECs treated with IL-1ß or actinomycin D was 8%, 40% and 44%, respectively, relative to the level of p38 MAPK phosphorylation in HUVECs treated with a mixture of IL-1ß and actinomycin D. Z-VAD-FMK did not affect p38 MAPK activation in HUVECs treated with IL-1ß in the presence of actinomycin D. The level of p38 MAPK phosphorylation in the presence of SB239063 was 42% of that found in HUVECs treated with IL-1ß in the presence of actinomycin D. Data shown are the averages and standard deviations of three Western Blot analyses. Pound key marks statistically insignificant effect of Z-VAD-FMK on p38 MAPK phosphorylation (p-value >0.05). Asterisks mark statistically significant differences (p-value <0.05) in p38 MAPK phosphorylation in comparison with p38 MAPK phosphorylation in HUVECs treated with IL-1ß in the presence of actinomycin D. ***Panel C*** shows the effect of SB239063 on MSC adhesion to HUVECs. HUVECs were treated with a mixture of 10 ng/ml IL-1ß and 10 µg/ml actinomycin D (ActD) in the presence and absence of 50 µM SB239063. In the presence of SB239063 MSC adhesion was indistinguishable from the adhesion to untreated HUVECs (marked with a pound key, p-value >0.05). Combination of SB239063 and 50 µM Z-VAD-FMK had no further effect on the MSC adhesion and was not significantly different from MSC adhesion to untreated HUVECs (marked with a pound key, p-value >0.05). Data shown are the averages and standard deviations of five adhesion assays. Asterisk marks statistically significant 3.4-fold increase (p-value <0.05) in the MSC adhesion in comparison with the adhesion to untreated HUVECs.

Apoptosis may coincide with an activation of stress dependent protein kinases including p38 MAPK [16]. Previously, we have shown that endothelial stress activates p38 MAPK in HUVECs. At least in part, this activation is mediated by von Willebrand factor that is secreted by stressed ECs and mediates EC adhesiveness for MSCs via activation of p38 MAPK [6], [17]. In order to elucidate the role of p38 MAPK in the regulation of MSC adhesion to apoptotic ECs, HUVECs were treated with IL-1ß, actinomycin D or a mixture of IL-1ß and actinomycin D in the presence and absence of p38 MAPK inhibitor SB239063. IL-1ß, actinomycin D and a mixture of IL-1ß and actinomycin D activated p38 MAPK in HUVECs (Fig. 7A). Activation of p38 MAPK was quantified after acquisition of digital images of the Western blots on a Kodak Image Station (Fig. 7B). The level of p38 MAPK phosphorylation in non-stimulated HUVECs was 7% of the level of phosphorylation in cells treated with IL-1ß in the presence of actinomycin D. Phosphorylation of p38 MAPK in HUVECs treated with IL-1ß or actinomycin D was 40 and 44%, respectively, of the level of p38 MAPK phosphorylation in cells treated with IL-1ß in the presence of actinomycin D. Z-VAD-FMK had no significant effect on p38 MAPK phosphorylation in HUVECs treated with IL-1ß in the presence of actinomycin D. This suggests that caspase activity is not required for p38 MAPK phosphorylation. SB239063 reduced p38 MAPK phosphorylation 2.5-fold in cells treated with IL-1ß in the presence of actinomycin D. Since SB239063 is a competitive inhibitor of p38 MAPK, the last observation suggests that the binding of the inhibitor with the active site of p38 MAPK allosterically inhibits p38 MAPK phosphorylation.

Inhibition of p38 MAPK with SB239063 completely abrogated the stimulation of MSC adhesion to HUVECs treated with IL-1ß in the presence of actinomycin D (Fig. 7C), while the activation of caspase-3/7 was not inhibited (Fig. 8). These results suggest that p38 MAPK is a primary regulator of EC adhesiveness for MSCs while the activation of caspases potentiates the MSC adhesion.

## Discussion

Treatment of ECs with IL-1ß for 4 hours stimulated MSC adhesion 1.5 - fold (Fig. 2A) which was consistent with a limited response of human MSCs to activation of ECs with TNF-α or IL-1ß reported by us previously [6]. Others have reported 3-5-fold stimulation of the MSC adhesion after activation of ECs with IL-1ß or TNF-α, however, the period of time (24 hours) required to achieve such stimulation was substantially longer [18]. Experimental conditions employed in our studies were close to that used to study the adhesion of neutrophils and monocytes to HUVECs [11], [12]. At these conditions IL-1ß stimulated the adhesion of neutrophils 18-fold, while the adhesion of monocytes was stimulated 2.6-fold [12]. Overall the response of MSCs to activation of ECs with inflammatory factors was close to that displayed by monocytes.

Expression of E-selectin, ICAM-1 and VCAM-1 was upregulated on the cell surface of HUVECs treated with IL-1ß or TNF-α (Fig. 1). Cycloheximide, an inhibitor of protein synthesis, or actinomycin D, an inhibitor of RNA synthesis, inhibited the expression of E-selectin, ICAM-1 and VCAM-1 on the surface of HUVECs treated with IL-1ß or TNF-α (Fig. 1). Despite the inhibition of the cell surface expression of E-selectin, ICAM-1 and VCAM-1, the treatment of HUVECs with IL-1ß in the presence of actinomycin D stimulated MSC adhesion (Fig. 2A). Stimulation of the MSC adhesion by a mixture of IL-1ß and actinomycin D required 4 hours to fully develop (Fig. 2B) and resulted in a faster adhesion of MSCs to treated HUVECs (Fig. 2C).

Mitochondrial transmembrane potential of HUVECs was reduced by a treatment with a mixture of IL-1ß and actinomycin D (Fig. 3A). There was a strong correlation between the reduction of mitochondrial transmembrane potential of HUVECs and the adhesion of MSCs (R = 0.98, P-value = 0.003, Fig. 4) suggesting that MSC adhesion to ECs might be regulated by oxidative stress. Depolarization of the mitochondrial membrane decouples the electron transport chain of mitochondria resulting in accumulation of peroxides that participate in oxidation of lipids in the membrane of apoptotic cells [13]. Oxidized derivatives of arachidonic acid directly stimulate EC adhesiveness for monocytes [14], [19]. It was conceivable that MSC adhesion to apoptotic ECs is regulated in a similar way. Release of lipid mediators requires the action of lipoxygenase and phospholipase [14]. Here we tested the effects of baicalein, an inhibitor of lipoxygenase, and PACOCF3, an inhibitor of phospholipase A2, on MSC adhesion to apoptotic HUVECs (Fig. 6). Inhibition of phospholipase A2 and lipoxygenase had no effect on MSC adhesion to HUVECs treated with IL-1ß in the presence of actinomycin D. Oxidized fatty acids isolated from the membrane of apoptotic cells or apoptotic blebs are effective in the stimulation of monocyte adhesion to HUVECs [14], [20], [21], however, endogenous production of lipid mediators by apoptotic ECs might not be sufficient to support their activation. Indeed, the treatment of ECs with TNF-α or IL-1ß in the presence of cycloheximide or actinomycin D did not stimulate, but rather inhibited monocyte adhesion to HUVECs [11], [12].

Reduction of mitochondrial transmembrane potential is a strong indication of permeabilization of mitochondrial membrane leading to a leak of cytochrome C into the cytoplasm and the activation of caspase signaling cascade. Thus, the stimulation of MSC adhesion (Fig. 2B) occurred on a background of the mitochondrial failure (Fig. 3A) and apoptosis of ECs (Fig. 3B). We also tested whether the inhibition of caspases affects MSC adhesion to apoptotic ECs. Our data indicated that the treatment of HUVECs with a mixture of IL-1ß and actinomycin D in the presence of pan-caspase inhibitor Z-VAD-FMK reduced the stimulation of MSC adhesion by 60% (Fig. 5).

Previously we have shown that a variety of pro-apoptotic agents such as staurosporine, wortmannin and okadaic acid, as well as a mixture of IL-1ß or TNF-α and the inhibitors of RNA or protein synthesis stimulate MSC adhesion to ECs [6]. The adhesion of MSCs had a strong correlation with the secretion of von Willebrand factor by ECs (R = 0.86, *P*-value = 0.0003) [6]. In a separate study we found that von Willebrand factor may serve as an autocrine regulator of MSC adhesion to ECs acting via the activation of p38 MAPK of ECs [17]. P38-MAPK was found phosphorylated in HUVECs treated with IL-1ß in the presence of actinomycin D (Fig. 7A). Inhibition of caspases had no effect on the level of p38 MAPK phosphorylation (Fig. 7B) and, *vice versa*, the inhibition of p38 MAPK did not prevent caspase-3 activation in HUVECs (Fig. 8). Inhibition of p38 MAPK in HUVECs completely inhibited the stimulation of MSC adhesion by IL-1ß in the presence of actinomycin D (Fig. 7C). It is important to note that the level of MSC adhesion to HUVECs treated with a mixture of IL-1ß and actinomycin D in the presence Z-VAD-FMK was close to that obtained by the treatment of HUVECs with von Willebrand factor [17]. Considering that the inhibition of p38 MAPK completely abolished the stimulation of the MSC adhesion with a mixture of IL-1ß and actinomycin D we hypothesize that p38 MAPK is a primary regulator of EC adhesiveness for MSCs, while the activation of caspases potentiates the effect of p38 MAPK activation.

Overall, MSC adhesion to ECs in many aspects is similar to adhesion on monocytes such as the correlation with oxidative stress and the dependence on the activity of p38 MAPK and caspases. It remains to be investigated whether MSCs respond to the same lipid mediators as monocytes. Similarities between MSC and monocytes in their adhesion to ECs suggest similarities in their homing. Although a limited number of quantitative studies of the timing of MSC homing have been conducted, the results of these studies suggest that the effective time for MSC homing is 4–10 days after the injury [1], [2]. This period of time corresponds to the time of monocyte homing and clearance of apoptotic cells by macrophages. Data from *in vivo* studies are in agreement with *in vitro* models of MSC adhesion to apoptotic ECs, suggesting that “later homing” of MSCs might be a result of selective adhesion of MSCs to apoptotic ECs.
